# Leishmaniasis Incidence and *Leishmania* Species Distribution in the Apurímac, Ene and Mantaro River Valley, a High-Transmission Endemic Area of Peru

**DOI:** 10.3390/pathogens15080792

**Published:** 2026-07-25

**Authors:** Jime Rivera-Villar, Nyshon Rojas-Palomino, José Alarcón-Guerrero, Víctor Cárdenas-López, Rilder Gastelú-Quispe, Aide Sandoval-Juarez, Saúl Chuchón-Martínez

**Affiliations:** 1 Facultad de Ciencias Biológicas, Universidad Nacional San Cristóbal de Huamanga, Ayacucho 05001, Peru; jimi.rivera@unsch.edu.pe (J.R.-V.); jose.alarcon@unsch.edu.pe (J.A.-G.); victor.cardenas@unsch.edu.pe (V.C.-L.); rilder.gastelu@unsch.edu.pe (R.G.-Q.); saul.chuchon@unsch.edu.pe (S.C.-M.); 2 Centro Nacional de Salud Pública, Instituto Nacional de Salud, Lima 150108, Peru; asandoval@ins.gob.pe

**Keywords:** *Leishmania*, leishmaniasis, *Leishmania braziliensis*, nucleic acid denaturation, neglected diseases, tropical medicine, epidemiology

## Abstract

Background: Leishmaniasis remains a major public health problem in Peru, and the Apurímac, Ene and Mantaro River Valley (VRAEM) is an endemic area where, despite its epidemiological relevance, information on incidence and circulating *Leishmania* species remains limited. Methods: We estimated district-level cumulative incidence of leishmaniasis in the 60 districts of the VRAEM during 2015–2024, using national surveillance data and census population figures. In addition, molecular species typing was performed on 112 Giemsa-stained tissue smears obtained in health facilities located in Ayacucho districts belonging to the VRAEM region. Results: A total of 3589 cases were reported, yielding an estimated annual incidence rate of 80.01 cases per 100,000 inhabitants, which is 4.3-fold higher than the national average. Marked spatial heterogeneity was observed, with districts such as Pangoa, Río Tambo, Llochegua, and Pichari reporting estimated annual incidence rates above 130 cases per 100,000 inhabitants. Furthermore, of 55 samples processed by High-Resolution Melting Analysis, *Leishmania braziliensis* was found in 54.6% of samples, followed by *Leishmania guyanensis* in 21.8%. Conclusions: The VRAEM constitutes a high-transmission endemic focus of tegumentary leishmaniasis with marked inter-district heterogeneity and circulation of multiple *Leishmania* species, with *Leishmania braziliensis* as the predominant species. These findings support the need for targeted surveillance and species-informed clinical management strategies.

## 1. Introduction

Leishmaniasis, a disease caused by protozoa of the genus *Leishmania*, represents a major public health problem worldwide. In the Americas, it affects more than 52,000 people per year, with Brazil, Colombia, and Peru accounting for approximately 40%, 19%, and 13% of all reported tegumentary leishmaniasis cases on the continent, respectively [1]. Together with Afghanistan, Algeria, Costa Rica, Ethiopia, Iran, Sudan, and Syria, these countries report nearly 75% of all cutaneous leishmaniasis cases worldwide [2]. In addition, the progression of tegumentary leishmaniasis is associated with chronic lesions, disfigurement, social stigma, and substantial economic and psychosocial impact on affected patients [3].

In Peru, tegumentary leishmaniasis is widely distributed across multiple ecological regions, occurring in western Andean valleys at 800–3000 m above sea level (masl) and inter-Andean valleys at 1900–3200 masl [4], as well as in the highland rainforest at 400–1000 masl and the lowland rainforest at 80–400 masl [5].

According to the National Center for Epidemiology, Prevention and Control of Diseases, Ministry of Health of Peru, more than 164,000 leishmaniasis cases were reported between 2000 and 2024, with mucosal forms accounting for 6.6% of cases [6]. Over 65% of cases occur in males [6], mainly due to occupational activities such as agriculture and livestock farming, which increase exposure to the vector and disease transmission [7].

The disease in Peru is characterized by a high diversity of circulating *Leishmania* species. Up to eight species have been reported, including *Leishmania (Viannia) braziliensis*, *Leishmania (Viannia) peruviana*, *Leishmania (Viannia) guyanensis*, *Leishmania (Viannia) panamensis*, *Leishmania (Viannia) lainsoni*, *Leishmania (Viannia) shawi*, *Leishmania (Viannia) naiffi* and *Leishmania (Leishmania) amazonensis* [8,9,10,11,12,13].

In addition to the reported clinical forms such as localized cutaneous and mucosal leishmaniasis, less frequent presentations have been described, including disseminated cutaneous leishmaniasis (DCL) [7,14] and diffuse cutaneous leishmaniasis (DL) [15], the latter being particularly notable for its association with an exacerbated inflammatory response and poor response to conventional treatment [16].

In this context, identifying the infecting *Leishmania* species is clinically relevant, as it enables more specific post-treatment follow-up aimed at the early detection of reactivation or progression to aggressive forms, such as mucosal leishmaniasis. However, despite the recognized diversity of circulating species in the country, there is still a lack of studies integrating incidence data with species distribution in high-transmission endemic areas [17,18].

The Apurímac, Ene, and Mantaro River Valley (VRAEM) is located in the highland rainforest of Peru and currently encompasses 60 districts across the regions of Apurímac, Ayacucho, Huancavelica, Cusco, and Junín, with an economy primarily based on cacao, coffee, and coca leaf cultivation [19].

However, some areas of the VRAEM are affected by drug trafficking and associated violence, together with limited state presence, factors that have worsened living conditions and contributed to high rates of child malnutrition, inadequate public services and restricted access to healthcare. These conditions, together with intensive agricultural activity and frequent population movement, may sustain the transmission of tegumentary leishmaniasis and delay diagnosis and treatment in remote communities [20,21].

Although the diversity of *Leishmania* species in Peru has been widely documented and the VRAEM is recognized as an endemic area for the disease, data on incidence and circulating *Leishmania* species in this region remain limited. This gap restricts the identification of districts with the highest transmission risk and constrains the planning of targeted control measures. Therefore, this study aimed to estimate the incidence of tegumentary leishmaniasis in the VRAEM during 2015–2024, identify districts with elevated transmission, and determine the distribution of *Leishmania* species in clinical samples collected between 2018 and 2020 by health facilities in the Ayacucho districts of the VRAEM.

## 2. Materials and Methods

A cross-sectional observational study was conducted, integrating epidemiological surveillance data with molecular characterization of *Leishmania* species. For the incidence component, we estimated the annual incidence rates of tegumentary leishmaniasis in all districts of the VRAEM from 2015 to 2024, using publicly available national epidemiological situation data reported by the National Center for Epidemiology, Prevention and Control of Diseases, Ministry of Health of Peru. The laboratory component consisted of molecular typing of *Leishmania* species by high-resolution melting analysis (HRMA) from Giemsa-stained tissue smears collected between 2018 and 2020 at health facilities in the Ayacucho districts of the VRAEM.

All laboratory procedures were carried out at the Microbiology and Parasitology Laboratory of the Faculty of Biological Sciences, Universidad Nacional San Cristóbal de Huamanga.

### 2.1. Study Area

The VRAEM is located in the highland rainforest of Peru and comprises 60 districts across the departments of Apurímac, Ayacucho, Huancavelica, Cusco, and Junín, with altitudes ranging from 95 to 3840 masl and a total area of approximately 58,780 km^2^ [21].

Incidence analysis was conducted for all districts comprising the VRAEM. For *Leishmania* species typing, only samples from health facilities in VRAEM districts belonging to the Ayacucho region were included, mainly health centers and health posts in the provinces of Huanta and La Mar, as well as the Regional Hospital of Ayacucho, which receives referred patients from endemic areas within the study.

**Figure 1:**
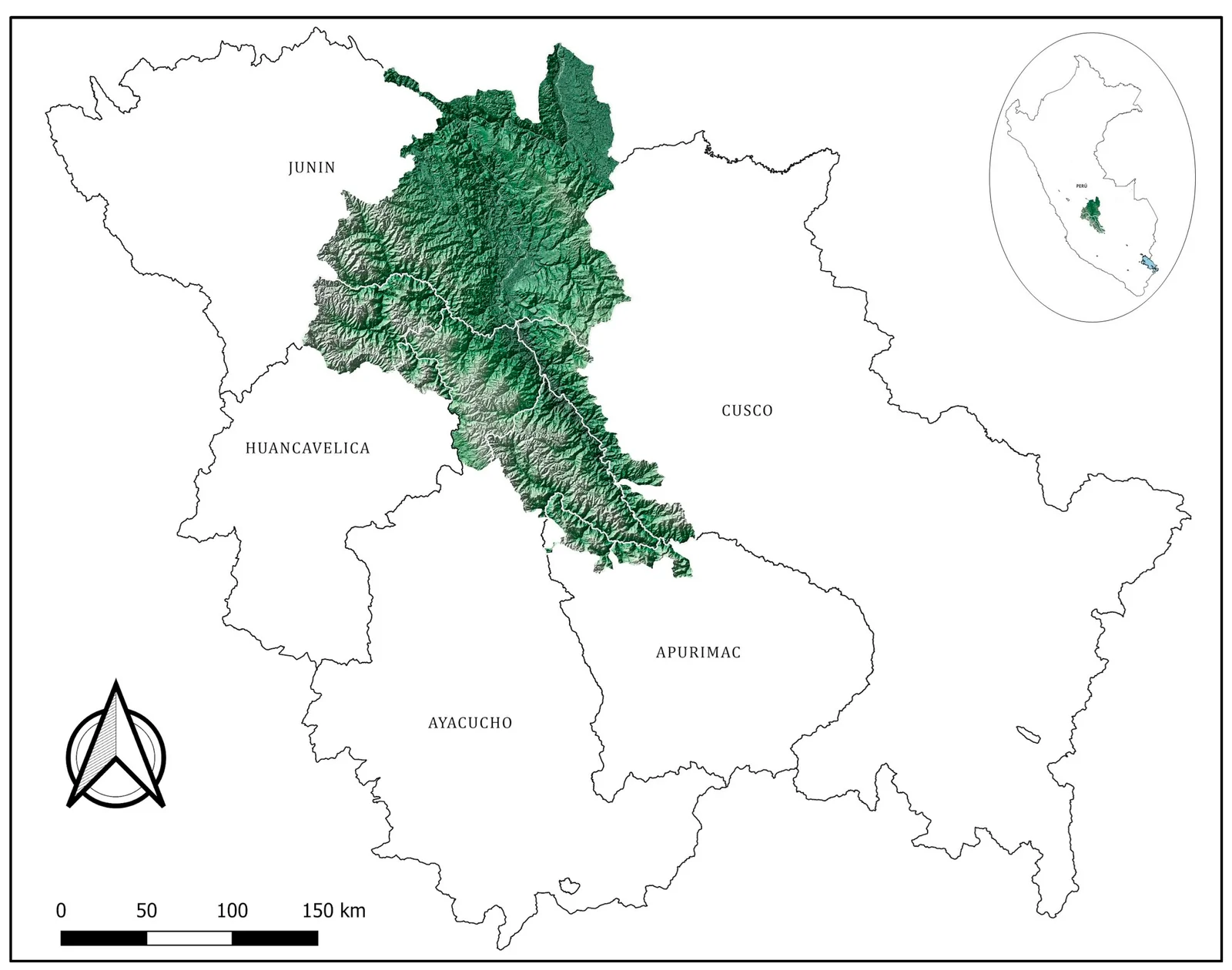
Map of the Apurímac, Ene, and Mantaro River Valley (VRAEM) in Peru, showing the constituent departments and a hillshade-based elevation model derived from SRTM data, which highlights the complex topography of the study area.

The Ayacucho districts of the VRAEM are located in the basin formed by the Apurímac, Ene, and Mantaro rivers, between 12°18′ and 13°22′ south latitude and 72°55′ and 74°17′ west longitude (Figure 1), covering an area of approximately 14,860.77 km^2^ and with altitudes ranging from 400 to 1900 masl. According to the Köppen classification, these areas have a tropical savanna climate (Aw) at lower altitudes and a temperate sub-humid mountain climate (Cwb) at higher elevations, with a dry season generally from May to September and higher rainfall between December and March.

### 2.2. Sample Collection

Giemsa-stained tissue smears fixed on glass slides and collected between 2018 and 2020 by health facilities within the San Francisco Health Network of Ayacucho were included. These slides had been obtained as part of the routine leishmaniasis diagnostic process using direct microscopic examination (DME).

We included Giemsa-stained tissue smears that yielded a positive result on DME and were obtained at any VRAEM health facility in Ayacucho, and excluded slides originating from areas or health facilities outside the VRAEM. The reporting health facility represents the location where the patient sought diagnosis and does not necessarily indicate the site of autochthonous transmission.

Tissue smear slides were re-examined under a bright-field microscope using immersion oil and a 100× objective and were categorized according to amastigote abundance using a cross-grading system [22], with the following categories: very low, low, moderate, and high presence of amastigotes.

### 2.3. Determination of Incidence

The incidence of tegumentary leishmaniasis in the VRAEM was estimated using data from the National Center for Epidemiology, Prevention and Control of Diseases, Ministry of Health of Peru, available at URL (accessed on 20 January 2026) https://www.dge.gob.pe/portalnuevo/sala-de-situacion/, which reports the number of notified cases at the district level for each epidemiological week. Weekly case data were obtained for the period 2015–2024 for all districts comprising the VRAEM across the regions of Apurímac, Ayacucho, Huancavelica, Cusco, and Junín.

Population data were obtained from the 2017 National Population and Housing Census and from the sociodemographic profile published by the National Institute of Statistics and Informatics (INEI–Peru), available at URL (accessed on 3 February 2026) https://censo2017.inei.gob.pe/resultados-definitivos-de-los-censos-nacionales-2017/.

To describe the overall burden of disease in each district, we first calculated the cumulative incidence rate by dividing the total number of notified cases during the 10-year study period (2015–2024) by the corresponding census population and expressing the result per 100,000 inhabitants. For each district, the cumulative number of cases was assumed to follow a Poisson distribution, and 95% confidence intervals for the cumulative incidence rates were estimated under this assumption.

To obtain an estimate of the average annual incidence, we derived the estimated annual incidence rate (EAIR) by dividing the cumulative incidence rate for the 10-year period by 10.

### 2.4. Leishmania Reference Strains

Infecting *Leishmania* species were identified by HRMA using DNA samples from promastigote forms of WHO reference strains. In addition, a non-reference WHO strain of *Leishmania (Viannia) peruviana* was included, as detailed in Table 1.

### 2.5. DNA Extraction

#### 2.5.1. DNA Extraction from Promastigotes

Cryovials containing the *Leishmania* reference strains were retrieved from liquid nitrogen storage, thawed at 37 °C, and processed for genomic DNA extraction using the PurelinkTM Genomic DNA Mini Kit (Ref. K1820–02, Invitrogen, Life Technologies, Carlsbad, CA, USA).

The samples were transferred to conical polypropylene tubes and centrifuged at 8000 rpm for 10 min. The supernatant was carefully removed and 180 µL of PureLinkTM Genomic Digestion Buffer (Ref. K1820–02, Invitrogen, Life Technologies, Carlsbad, CA, USA) was added to the pellet, followed by 20 µL of proteinase K. Samples were homogenized and incubated at 55 °C for two hours. Subsequently, lysis buffer and cold ethanol were added, and the mixture was centrifuged at 10,000 rpm for 1 min at room temperature. Samples were washed and eluted in a final volume of 50 µL.

The concentration of extracted DNA was determined using an Eon 5 spectrophotometer (BioTek Instruments, Winooski, VT, USA), and the A260/280 ratio was used as a purity parameter. DNA samples were stored at −20 °C until required.

#### 2.5.2. DNA Extraction from Slides

Giemsa-stained tissue smear slides were first submerged in methanol for 5 min to remove the Giemsa stain and immersion oil residues. Once dry and placed in a Petri dish, the biological material was recovered by scraping the slide surface with a scalpel blade.

For material recovery, 1 mL of methanol was added to the Petri dish, the biological material was collected and transferred to 1.5 mL conical polypropylene tubes, and then centrifuged at 8000 rpm for 10 min. The supernatant was discarded, and genomic DNA was extracted from the pellet following the same protocol as described for promastigotes. Extracted DNA was stored at −20 °C.

To assess DNA quality, samples were run on a 1.5% agarose gel. A single band was considered to indicate intact, non-degraded DNA, whereas multiple bands or a smear pattern was considered indicative of degradation. DNA concentration and purity were subsequently determined using an Eon5 spectrophotometer and the A260/280 ratio.

### 2.6. Cytochrome B Sequencing

Reference strains were confirmed by sequencing the cytochrome B gene, following the methodology reported by Foulet et al. (2007) [23], using primers Lei-Cyt B 09 (5′-TTATGGTGTAGGTTTTAGTYTAGGTT-3′) and Lei-Cyt B 12 (5′-TGCTAAAAAACCACTCATAAATATACT-3′).

### 2.7. Leishmania Identification by High-Resolution Melting Analysis (HRMA)

Extracted DNA samples were analyzed by real-time PCR targeting a conserved region of the kinetoplast minicircles (kDNA). The amplification reaction was performed in a final volume of 10 µL, using primers OL1 (5′-GGGGAGGGGCGTTCTGCGAA-3′) and OL2 (5′-CCGCCCCTATTTTACACCAACCCC-3′) at a concentration of 0.5 µM each [24].

The reaction mix contained 1X of Luminaris Color HRM Master Mix (Thermo Fisher Scientific, Waltham, MA, USA) with EvaGreen and 2 µL of genomic DNA normalized to 5 ng/µL. Cycling parameters were 95 °C for 10 min, followed by 35 cycles of 95 °C for 20 s, 57 °C for 30 s, and 72 °C for 1 min, with a final extension at 72 °C for 30 s. High-resolution melting was then performed with an initial denaturation at 95 °C for 15 s, followed by 60 °C for 1 min and a ramp at 0.8 °C/s up to 95 °C.

The analysis was performed using Rotor-Gene Q software v2.3.5 (Qiagen, Hilden, Germany). Genotyping was based on HRMA, with species-level typing performed by comparing the melting profiles of the samples with those of the reference strains included in this study. Samples showing >90% similarity to a reference profile were classified within the corresponding genotype, whereas samples with <90% similarity, or with profiles that did not correspond to any reference strain, were reported at the genus level as *Leishmania* spp.

### 2.8. Statistical Analysis

A database of leishmaniasis cases notified in the VRAEM districts during the 2015–2024 period was compiled. A chi-square test of homogeneity of proportions was performed with the null hypothesis of equal disease case proportions across districts, and the magnitude of association was quantified using Cramer’s V coefficient.

To identify districts with significant deviations in the number of reported cases relative to expected values under the homogeneity hypothesis, standardized adjusted residuals were examined, with an absolute value greater than 1.96 considered indicative of a significant contribution. All statistical analyses were performed using IBM SPSS Statistics version 27.0.1.0, with a 95% confidence level and a *p*-value ≤ 0.05 considered statistically significant.

Spatial maps of coca-leaf cultivation density and leishmaniasis incidence were generated using QGIS software, version 3.44.10. These maps were based on DEVIDA’s report “Perú. Monitoreo de cultivos de coca 2024” and on epidemiological data available from the National Center for Epidemiology, Prevention and Control of Diseases, Ministry of Health of Peru.

## 3. Results

During the period 2015–2024, a total of 58,394 cases of tegumentary leishmaniasis were notified in Peru, corresponding to an estimated annual incidence rate (EAIR) of 18.69 cases per 100,000 inhabitants. During the same period, 3589 cases were reported in the VRAEM, representing an EAIR of 80.01 cases per 100,000 inhabitants, which was heterogeneously distributed across districts in the regions of Apurímac, Ayacucho, Huancavelica, Cusco, and Junín. Districts with the highest case counts included Pangoa (727 cases), Río Tambo (702 cases), Llochegua (414 cases), Pichari (408 cases), Mazamari (266 cases), Sivia (158 cases), and Canayre (152 cases), all with EAIR values above 130 per 100,000 inhabitants, except Mazamari, which had an EAIR 74.47 cases per 100,000 inhabitants (Figure S1). In contrast, several districts in the Apurímac and Huancavelica regions had an EAIR below four cases of tegumentary leishmaniasis per 100,000 inhabitants.

A total of 112 Giemsa-stained tissue smear slides were collected from health facilities in the provinces of Huamanga (12/112; 10.7%), Huanta (54/112; 48.3%), and La Mar (46/112; 41.1%). Of these, 11 slides (9.8%) showed a very low amastigote concentration, ranging from a single amastigote in the entire smear to 1 amastigote per 100 fields; 83 slides (74.1%) presented a low concentration, defined as 2–10 amastigotes per field in at least 50 fields; 13 slides (11.6%) showed a moderate concentration, defined as 11–20 amastigote per field in at least 50 fields, and 5 slides (4.5%) exhibited a high concentration, defined as more than 21 amastigote per field in at least 10 fields (Table 2).

After nucleic acid extraction and DNA quality assessment, 55 of the 112 samples (49.1%) yielded adequate DNA for HRMA typing. Of these, 12.72% corresponded to imported cases and 87.28% to probable autochthonous cases; 43.64% were obtained from health facilities in Huanta province and 43.64% from health facilities in La Mar province.

**Figure 2:**
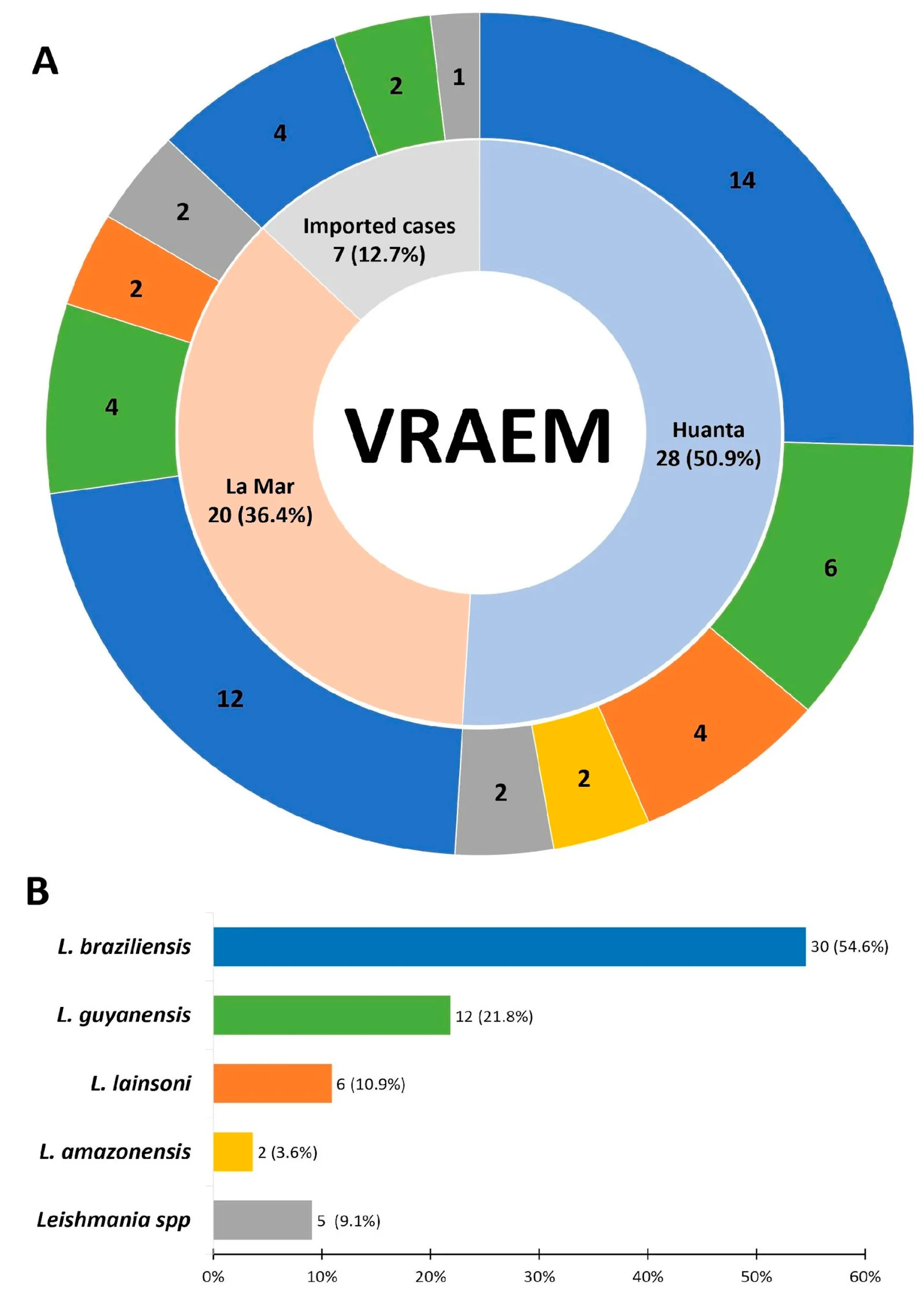
Species distribution of *Leishmania* identified in the VRAEM region. (**A**) Number and proportion of isolates obtained in health facilities from the provinces of Huanta and La Mar, and those corresponding to imported cases, with the outer ring indicating the distribution of *Leishmania* species within each group. (**B**) Overall frequency of each *Leishmania* species among all genotyped isolates, including samples classified as *Leishmania* spp. due to <90% similarity with any reference profile.

*Leishmania (Viannia) braziliensis* was identified as the predominant species in 30 samples (54.6%), followed by *Leishmania (Viannia) guyanensis* in 12 samples (21.8%), *Leishmania (Viannia) lainsoni* in 6 samples (10.9%), and *Leishmania (Leishmania) amazonensis* in 2 samples (3.6%). In contrast, 5 samples (9.1%) showed less than 90% similarity to any reference profile and were therefore reported at the genus level as *Leishmania* spp. (Figure 2).

At the district level, probable autochthonous transmission foci were identified in the districts of Canayre, Llochegua, Sivia, and Santillana in Huanta province, and in Ayna, Anco, and Santa Rosa in La Mar province. Canayre had the highest number of identified cases, followed by Anco and Llochegua (Figure S1). Imported cases originated from Cusco (9.1%), Junín (1.8%) and Huánuco (1.8%) (Table 3).

## 4. Discussion

In this study, the EAIR for the VRAEM was 80.01 per 100,000 inhabitants, more than four times higher than the national average of 18.69 per 100,000 inhabitants during the same period, indicating that the VRAEM constitutes a high-transmission focus for leishmaniasis in Peru. However, incidence was markedly heterogeneous across districts. While Pangoa, Río Tambo, Llochegua, and Pichari reported EAIRs exceeding 130 cases per 100,000 inhabitants, many districts, particularly in the regions of Apurímac and Huancavelica, had EAIRs below the national average (Table S1). This spatial heterogeneity is likely driven by geographic and ecological conditions, local economic activities, and differential population exposure to vector habitats [25,26].

The high incidence recorded in Pangoa and Río Tambo, in the Satipo province of Junín, as well as in Llochegua and Canayre in the Huanta province of Ayacucho, may be explained by the ecological characteristics of the highland rainforest, together with agriculture, deforestation, unplanned population growth, and limited healthcare presence. These conditions are favorable for phlebotomine sandfly activity and increased the risk of *Leishmania* transmission [27,28].

**Figure 3:**
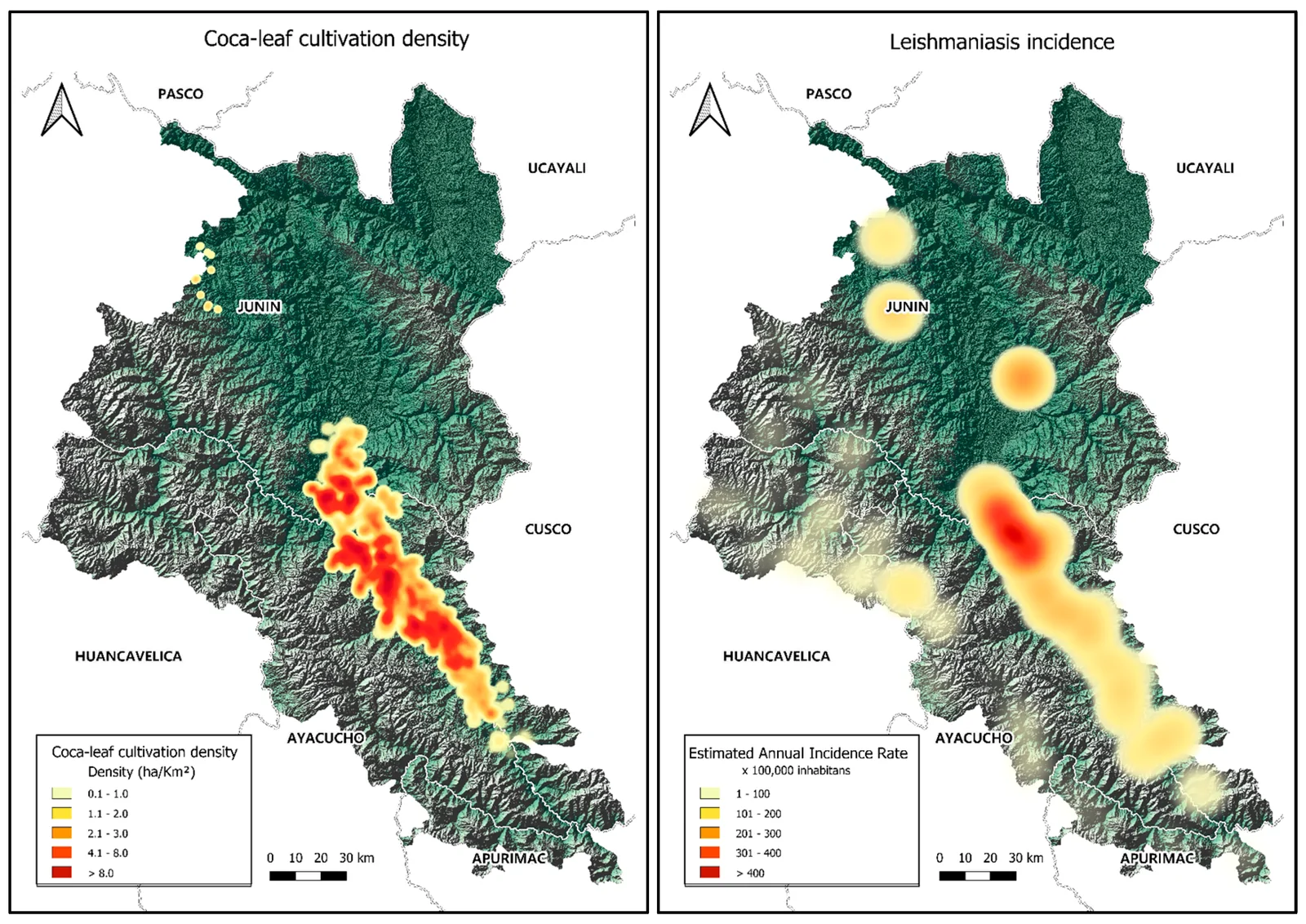
Spatial distribution of coca leaf cultivation density and estimated annual leishmaniasis incidence in the Apurímac, Ene, and Mantaro River Valley (VRAEM), Peru. (**Left**): Coca leaf cultivation density based on DEVIDA’s “Perú. Monitoreo de cultivos de coca 2024” report. (**Right**): Estimated annual incidence rate of leishmaniasis in the VRAEM, per 100,000 inhabitants.

In addition, this study found that localities with the highest leishmaniasis incidence in the VRAEM also coincide with areas of severe poverty, chronic malnutrition, persistent social conflict, illegal economies, drug trafficking [29,30], a high density of coca leaf cultivation [31] (Figure 3), and a limited presence of state institutions. These social determinants likely amplify the burden of disease by constraining access to healthcare services, delaying timely diagnosis, and hindering the provision of adequate treatment [32].

Although this was not a primary objective of the study, we observed that districts with the highest leishmaniasis incidence overlap with areas of intense coca leaf cultivation [31], reinforcing the hypothesis that limited state presence and restricted access to healthcare may play an important role in sustaining transmission in this region [32].

By comparison, the Madre de Dios region stands out as a highly endemic area, with an EAIR of more than 300 cases per 100,000 inhabitants [33], which is approximately 16 times higher than the national EAIR and 3.75 times higher than that of the VRAEM. In Madre de Dios, this elevated incidence coincides with extensive deforestation, unplanned population growth, limited healthcare coverage and widespread illegal mining activities [34]. Other regions with high transmission, with EAIR values comparable to the national average but lower than those observed in the VRAEM, include Amazonas, Ucayali, Pasco, Cusco, Cajamarca, San Martín and Huánuco [33].

With respect to molecular typing, in the present study a larger number of samples from Ayacucho were included and characterized by HRMA compared with previous studies. *Leishmania (Viannia) braziliensis* was the most frequently identified species (54.6%) and was detected in samples from health facilities in all three provinces, consistent with previous reports from jungle areas of departments such as Ayacucho, Cusco, and Junín [8,12].

Similar to the findings reported by De los Santos et al. (2024) [35], *Leishmania (Viannia) guyanensis* was identified in localities of Llochegua and Canayre. We also detected this species in the localities of Anco and Santillana, in the provinces of La Mar and Huanta, respectively. Two DNA samples from patients infected in Cusco but diagnosed in health facilities in Ayacucho were also identified as *Leishmania (Viannia) guyanensis*, in agreement with previous reports [8,9].

Other species were detected at lower frequency *Leishmania (Viannia) lainsoni* was identified mainly in samples from Canayre, in the Huanta province. Although this species was detected infrequently, it has been previously reported in Ayacucho and Cusco [8].

Finally, *Leishmania (Leishmania) amazonensis* was identified in 3.6% of samples, all of which were detected in Canayre. This species has been associated with diffuse cutaneous leishmaniasis, a severe and treatment-refractory clinical form of the disease [14,15,16]. Although its frequency was low, its identification in clinical samples from the VRAEM reinforces the importance of species-level typing as part of clinical management, particularly in atypical cases or those with poor therapeutic response. Conversely, *Leishmania (Viannia) peruviana*, a species frequently reported in Andean areas adjacent to the VRAEM, was not detected, which may reflect ecological differences, limited sample size, or lower circulation in the specific VRAEM localities sampled.

A proportion of samples (9.1%) could not be assigned to any reference profile because their similarity value was below 90%, and were therefore categorized as *Leishmania* spp. This loss of information was probably related to DNA quality issues in samples extracted from aged Giemsa-stained smears, residual oils or Giemsa, and the possible presence of species not included in the reference panel or even mixed infections.

An important limitation of this study was our inability to confirm *Leishmania* typing by sequencing, because amplification of the cytochrome B region was not achieved. In addition, DNA degradation in aged smears likely limited the representativeness of the subset typed by HRMA. Nevertheless, the sample size achieved is comparable to, or larger than, that of previous molecular surveys in Peru based on Giemsa-stained slides [36,37].

Despite these limitations, our findings are concordant with the spatial distribution previously reported for *Leishmania* species [8,9,12,33] as well as with the high incidence described in districts such as Llochegua, Sivia, Canayre and Anco [33].

Similar to other studies, we observed the co-existence of multiple *Leishmania* species within districts such as Canayre, Anco, and Ayna, which reflects a complex transmission setting where vector diversity and overlapping reservoir hosts may sustain multiple *Leishmania* transmission cycles simultaneously.

Regarding imported cases (12.7%), their origin from Cusco, Junín, and Huánuco reflects the well-documented mobility of populations within and around the VRAEM corridor, where agricultural workers, migrants, and others frequently move between departments. This mobility represents a recognized epidemiological challenge for control programs, since cases may be diagnosed far from their actual site of infection, potentially biasing local incidence estimates and complicating the targeting of interventions.

## 5. Conclusions

This study focused on *Leishmania* transmission in the VRAEM, estimated the annual incidence rate for this endemic area, and included species typing. In this context, we found a distribution of *Leishmania* species consistent with that expected for jungle areas. The predominance of *Leishmania (Viannia) braziliensis* was expected, as was the detection of *Leishmania (Viannia) guyanensis*, *Leishmania (Viannia) lainsoni*, and *Leishmania (Leishmania) amazonensis*. In contrast, we did not detect *Leishmania (Viannia) peruviana*.

Based on our estimates, the annual leishmaniasis rate in the VRAEM reaches 80.01 cases per 100,000 inhabitants, which is more than four times higher than the national average (18.69 cases per 100,000 inhabitants), highlighting the need to strengthen surveillance programs and generate better information to guide disease control strategies.

Future studies should incorporate prospective sample collection to avoid the limitations imposed by DNA degradation and integrate environmental and entomological data to better characterize transmission dynamics in this complex endemic area.

## Figures and Tables

**Table 1:** *Leishmania* reference strains used in the study.

WHO Reference Strain	Code
*Leishmania (Viannia) braziliensis*	MHOM/BR/75/M2904
*Leishmania (V.) peruviana*	MHOM/PE/84/LC26 *
*Leishmania (V.) guyanensis*	MHOM/BR/75/M4147
*Leishmania (V.) lainsoni*	MHOM/BR/81/M6426
*Leishmania (V.) panamensis*	MHOM/PA/71/LS94
*Leishmania (L.) amazonensis*	MHOM/BR/73/M2269
*Leishmania (L.) mexicana*	MNYC/BZ/62/M379

* This non-reference strain was used because no WHO reference strain is available for this species.

**Table 2:** Characteristics of tissue samples and transmission patterns by province of reporting health facility.

Province of Reporting Health Facility	Samples (*n* = 112)	Amastigote Abundance	DNA Quality	Transmission			
				IC		PAC	
Huamanga	12 (10.7%)	Low	Degraded	3	2.7%	—	—
			High-quality	—	—	6 *	5.4%
		Moderate	Degraded	2	1.8%	—	—
			High-quality		—	1 *	0.9%
Huanta	54 (48.2%)	Very low	Degraded	—	—	3	2.7%
			High-quality	1	0.9%	1	0.9%
		Low	Degraded	3	2.7%	23	20.5%
			High-quality	3	2.7%	12	10.7%
		Moderate	Degraded	—	—	1	0.9%
			High-quality	—	—	5	4.5%
		High	High-quality	—	—	2	1.8%
La Mar	46 (41.1%)	Very low	Degraded	—	—	4	3.6%
			High-quality	—	—	2	1.8%
		Low	Degraded	1	0.9%	16	14.3%
			High-quality	3	2.7%	13	11.6%
		Moderate	Degraded	—	—	1	0.9%
			High-quality	1	0.9%	2	1.8%
		High	High-quality	—	—	3	2.7%
			TOTAL	17	15.2%	95	84.8%

* The Huamanga province does not have active transmission; these values correspond to samples obtained from patients who live in endemic VRAEM areas of Ayacucho but were attended at health facilities within this jurisdiction. IC: Imported cases; PAC: Probable autochthonous cases.

**Table 3:** Spatial and genotypic distribution of *Leishmania* isolates by provincial health jurisdiction and probable location of infection.

Provincial Health Jurisdiction	District of Sample Origin	Genotype	Classification		Probable Location of Infection
			PAC	IC	
Huamanga	Ayacucho	*L. braziliensis*	1	—	Huanta—Llochegua
			1	—	La Mar—Ayna
			1	—	La Mar—Anco
			1	—	Huanta—Canayre
		*L. guyanensis*	1	—	La Mar—Anco
		*L. lainsoni*	1	—	Huanta—Canayre
		*L. amazonensis*	1	—	Huanta—Canayre
	**Subtotal**		**7 (12.72%)**		
Huanta	Canayre	*L. guyanensis*	1	—	Huanta—Canayre
		*L. amazonensis*	1	—	Huanta—Canayre
	Huanta	*L. braziliensis*	—	3	Cusco
			1	—	Huanta—Sivia
			2	—	Huanta—Llochegua
			1	—	La Mar—Santa Rosa
			1	—	Huanta—ND
	Llochegua	*L. braziliensis*	1	—	Huanta—Llochegua
			3	—	Huanta—ND
		*L. guyanensis*	4	—	Huanta—ND
	Santillana	*L. braziliensis*	1	—	Huanta—ND
		*L. lainsoni*	1	—	Huanta—ND
		*L. guyanensis*	1	—	Huanta—Santillana
	Sivia	*L. braziliensis*	3	—	Huanta—ND
	**Subtotal**		**24 (43.64%)**		
La Mar	Anchihuay	*Leishmania* spp.	—	1	Satipo—Junín
	Anco	*L. braziliensis*	2	—	La Mar—Anco
		*L. guyanensis*	—	1	Cusco
			1	—	La Mar—Anco
		*L. lainsoni*	1	—	Huanta—Canayre
	Ayna	*L. braziliensis*	3	—	La Mar—ND
		*L. guyanensis*	—	1	Cusco
			1	—	La Mar—ND
		*L. lainsoni*	1	—	La Mar—ND
		*Leishmania* spp.	1	—	La Mar—ND
			1	—	La Mar—Ayna
			1	—	Huanta—Canayre
	Santa Rosa	*L. braziliensis*	—	1	Huánuco
			3	—	La Mar—ND
			1	—	La Mar—Ayna
		*L. guyanensis*	1	—	La Mar—ND
		*L. lainsoni*	1	—	La Mar—ND
		*Leishmania* spp.	1	—	Huanta—Sivia
	Tambo	*L. lainsoni*	1	—	Huanta—Canayre
	**Subtotal**		**24 (43.64%)**		

IC: Imported cases; PAC: Probable autochthonous cases; ND: Not determined.

## Data Availability

The original contributions presented in this study are included in the article/supplementary material. Further inquiries can be directed to the corresponding author.

## Supplementary Materials

The following supporting information can be downloaded at: https://www.mdpi.com/article/10.3390/pathogens15080792/s1, Figure S1: Estimated annual incidence rates of tegumentary leishmaniasis in the VRAEM by districts for the period 2015–2024. The solid red line represents the national estimated annual incidence rates; the dashed line represents the VRAEM estimated annual incidence rate; Table S1: Estimated annual incidence rates.
