# Supplementary material for: Leishmaniasis Incidence and Leishmania Species Distribution in the Apurímac, Ene and Mantaro River Valley, a High-Transmission Endemic Area of Peru
Source: Pathogens. 2026 Jul 25;15(8):792. doi: 10.3390/pathogens15080792 (PMC13517079; doi:10.3390/pathogens15080792)
Supplement: Supplementary file 1 [file pathogens-15-00792-s001.zip › Table S1. Estimated annual incidence rates.pdf]

Table S1. Estimates annual incidence rates

REGIÓN	PROVINCE	DISTRICT	2017 census population	2015		2016		2017		2018		2019		2020		2021		2022		2023		2024		Total cases	Estimated cumulative incidence rate (per 100,000 inhabitants)	95% confidence interval	Estimated annual incidence rate (cumulative incidence rate/10)
				TL	IR	TL	IR	TL	IR	TL	IR	TL	IR	TL	IR	TL	IR	TL	IR	TL	IR	TL	IR				
**PERÚ**			**31237385**	**6002**	**19.27**	**7437**	**23.62**	**6664**	**20.94**	**6144**	**19.1**	**5425**	**16.68**	**4740**	**14.53**	**5473**	**16.56**	**5762**	**17.33**	**5424**	**16.03**	**5323**	**15.6**	**58394**	186.94	(185.42 - 188.45)	**18.69**
**VRAEM**			**448582**	**323**	—	**447**	—	**376**	—	**434**	—	**371**	—	**328**	—	**399**	—	**307**	—	**317**	—	**287**	—	**3589**	800.08	(773.90 - 826.25)	**80.01**
	**ANDAHUAYLAS**	ANDARAPA	5459	0	0	0	0	0	0	0	0	2	30.81	0	0	0	0	0	0	0	0	0	0	2	36.64	(0.00 - 87.41)	3.66
		KAQUIABAMBA	1816	0	0	0	0	0	0	0	0	0	0	0	0	0	0	0	0	0	0	0	0	0	0		0
		PACOBAMBA	3452	0	0	0	0	0	0	1	20.6	0	0	0	0	0	0	0	0	0	0	1	33.09	2	57.94	(0.00 - 138.23)	5.79
		EL PORVENIR	1728	0	0	0	0	0	0	0	0	0	0	0	0	0	0	0	0	0	0	0	0	0	0	—	0
		HUACCANA	6634	0	0	0	0	2	19.2	1	9.56	0	0	0	0	0	0	0	0	0	0	0	0	3	45.22	(0.00 - 96.39)	4.52
	**CHINCHEROS**	OCOBAMBA	6759	0	0	2	23.94	0	0	2	23.75	1	11.82	0	0	0	0	0	0	0	0	1	14.78	6	88.77	(17.74 - 159.80)	8.88
		ONGOY	2344	0	0	0	0	0	0	1	10.82	0	0	0	0	0	0	0	0	0	0	0	0	1	42.66	(0.00 - 126.28)	4.27
		ROCCHACC	2689	0	0	0	0	0	0	0	0	0	0	0	0	0	0	0	0	0	0	0	0	0	0	—	0
		AYAHUANCO	1196	1	6.9	1	16.68	1	16.5	2	32.66	1	16.16	0	0	0	0	1	92.59	0	0	1	85.98	8	668.90	(205.37 - 1132.42)	66.89
		CANAYRE	3520	1		14	460.37	12	390.37	29	933.68	30	956.02	9	214.54	15	334.6	13	303.53	11	248.93	18	393.87	152	4318.18	(3631.69 - 5004.67)	431.82
		CHACA	2162	0	0	0	0	0	0	0	0	0	0	0	0	0	0	0	0	0	0	0	0	0	0	—	0
		LLOCHEGUA	10058	50	355.95	49	438.09	23	203.45	57	498.95	59	511	33	280.8	45	388.63	29	252.83	30	258.84	39	344.07	414	4116.13	(3719.63 - 4512.63)	411.61
		PUCACOLPA	2523	0	0	0	0	0	0	1	12.17	0	0	1	35.75	1	4.5	0	0	0	0	0	0	3	118.91	(0.00 - 253.46)	11.89
	**HUANTA**	SANIRLLANA	3852	0	0	1	13.52	0	0	0	0	1	19.92	1	23.98	3	77.26	0	0	1	28.01	0	0	7	181.72	(47.10 - 316.35)	18.17
		SIVIA	10797	22	178.21	20	137.04	13	88.13	24	161	9	59.73	8	64.06	21	180.01	12	109.1	10	88.1	19	174.42	158	1463.37	(1235.19 - 1691.55)	146.34
		UCHURACCAY	3522	0	0	0	0	0	0	0	0	0	0	0	0	0	0	0	0	0	0	0	0	0	0	—	0
		HUAMANGUILLA	4162	0	0	0	0	0	0	0	0	0	0	0	0	0	0	0	0	0	0	0	0	0	0	—	0
		HUANTA	39517	3	6.33	1	2.46	2	4.87	3	7.23	3	7.16	2	4.65	1	2.29	0	0	2	4.55	0	0	17	43.02	(22.57 - 63.47)	4.30
**AYACUCHO**		IGUAÍN	2667	0	0	0	0	0	0	0	0	0	0	0	0	0	0	0	0	0	0	0	0	0	0	—	0
		LURICOCHA	5490	0	0	0	0	0	0	0	0	0	0	0	0	1	15.92	0	0	0	0	0	0	1	18.21	(0.00 - 53.92)	1.82
		ANCHIHUAY	4039	0	0	4	70	4	69	7	120	2	34	1	21	1	23	0	0	1	24	5	130	25	618.97	(376.33 - 861.60)	61.90
		ANCCO	7969	10	59	9	79	6	52	10	86	9	77	4	46	2	25	9	159	7	126	4	75	70	878.40	(672.62 - 1084.18)	87.84
		AYNA	9298	18	170	21	197	14	130	17	156	9	82	11	106	10	100	18	186	15	154	10	106	143	1537.97	(1285.89 - 1790.04)	153.80
		CHUNGUI	4218	1	14	4	54	4	54	1	13	6	85	1	21	5	113	3	69	3	70	2	49	30	711.24	(456.72 - 965.75)	71.12
	**LA MAR**	SAMUGARI	9410	10	127	12	151	6	75	11	136	4	49	3	27	5	45	7	78	5	57	4	46	67	712.01	(541.52 - 882.50)	71.20
		SANTA ROSA	11279	8	71	13	114	15	130	14	120	9	76	15	116	15	117	6	48	7	56	3	25	105	930.93	(752.87 - 1109.00)	93.09
		CHILCAS	1643	0	0	0	0	0	0	1	32	0	0	0	0	0	0	0	0	0	0	0	0	1	60.86	(0.00 - 180.16)	6.09
		LUIS CARRANZA	1278	0	0	0	0	0	0	0	0	0	0	0	0	0	0	1	74	0	0	0	0	1	78.25	(0.00 - 231.61)	7.82
		SAN MIGUEL	10326	2	21	2	21	0	0	1	10	1	10	0	0	1	9	1	13	3	37	0	0	11	106.53	(43.57 - 169.48)	10.65
		TAMBO	10173	0	0	0	0	2	10	0	0	0	0	0	0	0	0	0	0	0	0	0	0	2	19.66	(0.00 - 46.91)	1.97
		INKAWASI	4285	0	0	1	19.67	1	19.57	1	19.46	0	0	2	39.53	5	98.33	2	39.79	0	0	0	0	12	280.05	(121.60 - 438.50)	28.00
		KIMBIRI	15962	16	94.87	22	129.69	14	82.07	13	75.81	3	19.93	9	46.08	3	15.46	12	86.06	13	93.24	9	62.21	114	714.20	(583.09 - 845.30)	71.42
**CUSCO**	**LA CONVENCION**	PICHARI	22691	35	172.28	56	274.05	37	180.07	46	222.67	50	240.67	38	142.76	52	186.29	27	116.78	31	131.72	36	151.82	408	1798.07	(1623.59 - 1972.54)	179.81
		VILLA KINIRARINA	1974	0	0	0	0	0	0	3		1	45.62	1	41.44	0	0	2	79.81	0	0	0	0	7	354.61	(91.91 - 617.31)	35.46
		VILLA VIRGEN	1980	0	0	2	109.41	3	163.4	3	162.34	6	322.93	0	0	1	41.53	0	0	4	165.77	1	41.79	20	1010.10	(567.40 - 1452.80)	101.01
		CHINCHIHUASI	1979	0	0	0	0	0	0	0	0	0	0	0	0	0	0	2	112.68	0	0	0	0	2	101.06	(0.00 - 241.12)	10.11
	**CHURCAMPA**	PAUCARBAMBA	4346	0	0	0	0	0	0	0	0	1	33.66	0	0	0	0	0	0	1	58.31	0	0	2	46.02	(0.00 - 109.80)	4.60
		PACHAMARCA	1110	0	0	0	0	0	0	0	0	1	33.66	0	0	0	0	0	0	1	58.31	0	0	2	180.18	(0.00 - 429.90)	18.02
		SAN PEDRO DE CORIS	3445	0	0	0	0	0	0	0	0	0	0	0	0	0	0	0	0	0	0	0	0	0	0	—	0
		ANDAYMARCA	1393	0	0	0	0	0	0	0	0	0	0	1	39.15	0	0	1	38.6	0	0	0	0	2	143.58	(0.00 - 342.56)	14.36
		HUACHOCOLPA	1875	0	0	0	0	1	15.68	0	0	0	0	0	0	0	0	1	42.03	0	0	0	0	2	106.67	(0.00 - 254.50)	10.67
		ROBLE	1300	0	0	0	0	0	0	0	0	0	0	0	0	0	0	0	0	0	0	0	0	0	0	—	0
		SURCUBAMBA	4601	0	0	0	0	0	0	0	0	0	0	0	0	0	0	0	0	0	0	0	0	0	0	—	0
		IRNTAY PUNCU	3010	0	0	0	0	0	0	0	0	0	0	0	0	0	0	0	0	0	0	1	64.31	1	33.22	(0.00 - 98.34)	3.32
		ACOSTAMBO	3266	0	0	0	0	0	0	0	0	0	0	0	0	0	0	0	0	0	0	0	0	0	0	—	0
		ACRAQUIA	3522	0	0	0	0	0	0	0	0	0	0	0	0	0	0	0	0	0	0	0	0	0	0	—	0
**HUANCAVELICA**		AHUAYCHA	3873	0	0	0	0	0	0	0	0	0	0	0	0	1	25.36	0	0	0	0	0	0	1	25.82	(0.00 - 76.43)	2.58
		COLCABAMBA	7106	0	0	0	0	2	15.93	0	0	2	15.71	0	0	0	0	0	0	0	0	1	9.31	5	70.36	(8.69 - 132.04)	7.04
	**TAYACAJA**	DANIEL HERNANDEZ	9089	0	0	0	0	0	0	0	0	0	0	0	0	0	0	0	0	0	0	0	0	0	0	—	0
		HUARIBAMBA	1988	0	0	0	0	0	0	1	12.47	0	0	0	0	0	0	0	0	0	0	0	0	1	50.30	(0.00 - 148.89)	5.03
		ÑAHUIMPUQUIO	1480	0	0	0	0	0	0	0	0	0	0	0	0	0	0	0	0	0	0	0	0	0	0	—	0
		QUICHUAS	2288	0	0	0	0	0	0	1	23.62	0	0	0	0	0	0	0	0	1	23.74	1	25.94	3	131.12	(0.00 - 279.49)	13.11
		QUISHUAR	731	0	0	0	0	0	0	0	0	0	0	0	0	0	0	0	0	0	0	0	0	0	0	—	0
		PAMPAS	6678	0	0	0	0	0	0	0	0	2	17.41	1	9.47	0	0	0	0	0	0	0	0	3	44.92	(0.00 - 95.76)	4.49
		PAZOS	3358	0	0	0	0	1	13.64	0	0	1	13.45	1	9.47	0	0	0	0	0	0	0	0	3	89.34	(0.00 - 190.44)	8.93
		PICHOS	1914	0	0	0	0	0	0	0	0	0	0	0	0	0	0	0	0	0	0	0	0	0	0	—	0
		SALCABAMBA	3828	0	0	0	0	0	0	0	0	0	0	0	0	0	0	1	26.69	0	0	0	0	1	26.12	(0.00 - 77.32)	2.61
		SALCAHUASI	2616	0	0	0	0	0	0	0	0	0	0	0	0	0	0	0	0	0	0	0	0	0	0	—	0
		SAN MARCOS DE ROCCHAC	1813	0	0	0	0	0	0	0	0	0	0	0	0	0	0	0	0	0	0	0	0	0	0	—	0
	**CONCEPCION**	ANDAMARCA	3502	0	0	0	0	0	0	0	0	0	0	1	26.06	0	0	0	0	0	0	0	0	1	28.56	(0.00 - 84.52)	2.86
	**HUANCAYO**	PARIAHUANCA	5130	0	0	0	0	0	0	0	0	1	16.36	1	17.51	1	19.36	0	0	0	0	1	16.04	4	77.97	(1.56 - 154.39)	7.80
		STO DOMINGO DE ACOBAMBA	6222	1	12.92	1	12.83	0	0	1	12.65	1	12.56	0	0	0	0	0	0	0	0	0	0	4	64.29	(1.29 - 127.29)	6.43
**JUNIN**		MAZAMARI	35719	22	36.53	33	52.81	33	52.43	26	41.02	22	34.46	20	47.91	37	84.29	28	66.48	25	56.63	20	44.71	266	744.70	(655.21 - 834.20)	74.47
	**SAIRPO**	PANGOA	54240	73	115.78	115	186.48	89	143.29	76	121.5	67	112.86	71	112.23	71	114.44	52	77.79	63	92.21	50	72.08	727	1340.34	(1242.91 - 1437.77)	134.03
		RIO TAMBO	26036	50	85.59	64	108.78	89	150.19	75	125.68	62	103.15	86	283.74	90	299.83	66	213.98	71	234.25	49	164.82	702	2696.27	(2496.81 - 2895.72)	269.63
		VIZCATAN DEL ENE	4252	0	0	0	0	2		5		5	137.59	7	140.85	12	199.04	13	225.38	12	195.89	11	171.66	67	1575.73	(1198.42 - 1953.04)	157.57

TL: Tegumentary Leishmaniasis

IR: Incidence rate per 100,000 inhabitants.

Estimated cumulative incidence rate (per 100,000 inhabitants) = (total number of cases during 2015–2024 / 2017 census population) × 100,000

Estimated annual incidence rate = Estimated cumulative incidence rate / 10 (2015–2024 period divided by 10 years
